# Atlas of Syphilis and the Venereal Diseases

**Published:** 1900-03

**Authors:** 


**Atlas of Syphilis and the Venereal Diseases.** By Dr. Franz Mracek. Authorised translation from the German. Edited by L. Bolton Bangs, M.D. Pp. 16, 122. London: The Rebman Publishing Co. (Ltd). Philadelphia: W. B. Saunders. 1898.

The seventy-one full-page coloured plates have been selected to illustrate those forms of venereal disease which are of frequent occurrence and of practical importance, only those which are rarely seen and are chiefly of interest to the specialist being omitted. They are reproductions of water-colour drawings taken from actual cases, and a brief outline of the clinical history of the patient is given with each. In addition, 116 pages at the end of the work are devoted to a condensed systematic account of the disorders which have been illustrated, and of their treatment. We would suggest that the numerous prescriptions given would be more useful to the British practitioner if the proportions of the active ingredients were given uniformly to a total quantity of the preparation of ℥j. or Oj., rather than to irregular quantities like ʒijss., ℥ij., ℥vj., and O ij.

The plates are of excellent quality, and appear to us to illustrate their subjects as well as any plates could do so. We believe they will prove of much use to those practitioners who have few opportunities of examining actual cases, and to students who have their experience yet to acquire. The handy size of the atlas is a very commendable feature.

